# Evolutionary walks through flower colour space driven by gene expression in *Petunia* and allies (Petunieae)

**DOI:** 10.1098/rspb.2023.0275

**Published:** 2023-07-12

**Authors:** Lucas C. Wheeler, Amy Dunbar-Wallis, Kyle Schutz, Stacey D. Smith

**Affiliations:** Department of Ecology and Evolutionary Biology, University of Colorado, 1900 Pleasant Street 334 UCB, Boulder, CO 80309-0334, USA

**Keywords:** pigmentation, flavonoid, methylation, trade-offs, gene regulation, convergent evolution

## Abstract

The structure and function of biochemical and developmental pathways determine the range of accessible phenotypes, which are the substrate for evolutionary change. Accordingly, we expect that observed phenotypic variation across species is strongly influenced by pathway structure, with different phenotypes arising due to changes in activity along pathway branches. Here, we use flower colour as a model to investigate how the structure of pigment pathways shapes the evolution of phenotypic diversity. We focus on the phenotypically diverse Petunieae clade in the nightshade family, which contains *ca* 180 species of *Petunia* and related genera, as a model to understand how flavonoid pathway gene expression maps onto pigment production. We use multivariate comparative methods to estimate co-expression relationships between pathway enzymes and transcriptional regulators, and then assess how expression of these genes relates to the major axes of variation in floral pigmentation. Our results indicate that coordinated shifts in gene expression predict transitions in both total anthocyanin levels and pigment type, which, in turn, incur trade-offs with the production of UV-absorbing flavonol compounds. These findings demonstrate that the intrinsic structure of the flavonoid pathway and its regulatory architecture underlies the accessibility of pigment phenotypes and shapes evolutionary outcomes for floral pigment production.

## 1. Introduction

Biologists have long observed that species are not uniformly distributed across the space of possible phenotypes, but are clustered in certain regions of the space, leaving gaps in others. One explanation for this pattern is natural selection, where the clusters represent phenotypes associated with some adaptive optimum (e.g. [1,2]). Another contributing factor may be developmental bias, where some phenotypes are more likely outcomes given the underlying genetic and developmental pathways and others are inaccessible [3,4]. As selection acts upon the products of development, these forces may also act in concert and jointly contribute to the patchiness of phenotype space [5].

While much of our understanding of the factors shaping phenotype space comes from experimental work (e.g. [6–8]), macroevolutionary approaches can also provide unique insights. For example, macroevolutionary trends may mirror ontogenetic trajectories, suggesting that phenotypic evolution is biased by developmental processes [9]. Comparative studies can also be used to estimate the degree of phenotypic integration, which is tied to stronger developmental bias [10]. Beyond purely morphological studies, the field of evo-devo has uncovered numerous instances of the same genes and pathways underlying independent origins of complex traits in distantly related lineages (e.g. [11,12]), highlighting the central role of genetic and developmental pathways in shaping evolutionary trajectories.

Here, we use flower colour as a model system to interrogate the relationship between pathway structure and phenotypic diversity at a macroevolutionary scale. The developmental basis for flower pigmentation, in particular through anthocyanin production, is arguably one of the best understood pathways in plants and is widely conserved across species [13,14]. With an extensive foundation in the genetics of anthocyanin biosynthesis, the mechanisms responsible for flower colour evolution have been dissected in a diverse and growing list of taxa (e.g. [15–20]). Together these studies suggest that while changes in enzyme function can contribute to flower colour transitions (e.g. [21,22]), differences in gene expression are by far the predominant mode of colour macroevolution [23,24]. Nevertheless, we lack a broader understanding of how the structure of the pathway combines with differential gene expression to give rise to the range of observed flower pigment phenotypes and possibly explain those that are not observed [25].

**Figure 1.**
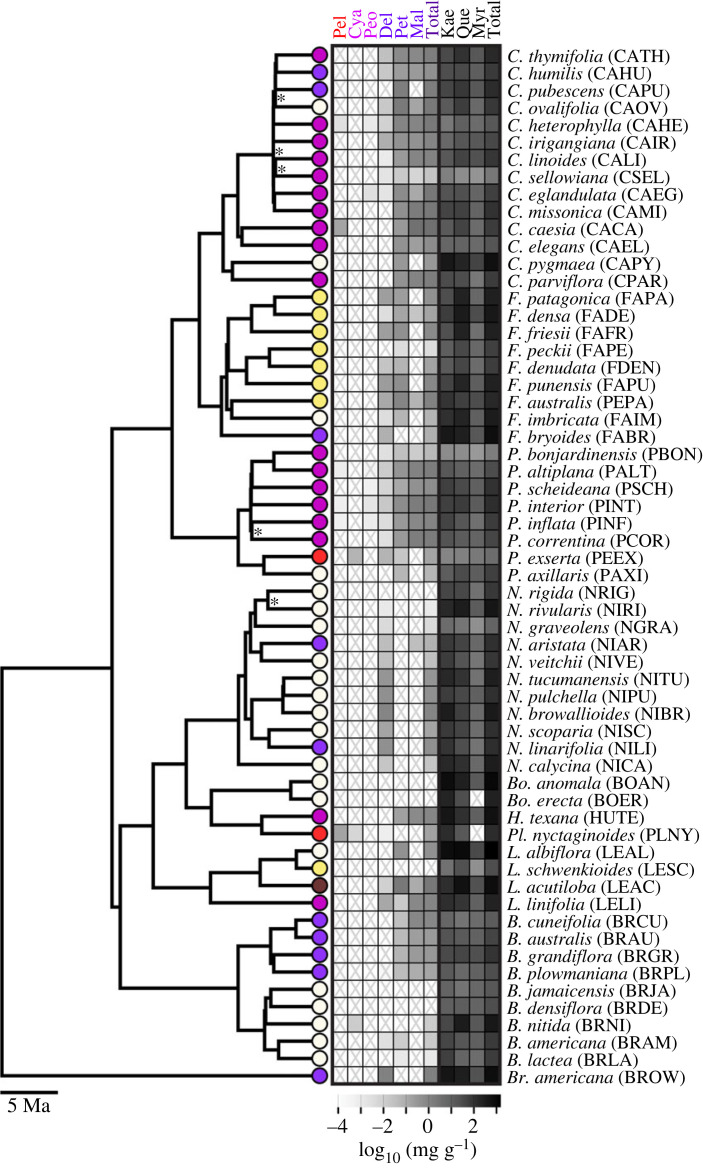
Flavonoid pigmentation varies across the Petunieae clade. Species tree for 60 taxa from Astral analysis of 3672 gene trees. Nodes with less than 0.95 local posterior support are indicated with asterisks. A full species tree with all supports annotated is shown in electronic supplementary material, figure S1. The tree is rooted with *Browallia americana* as the outgroup. Flower colours (white, yellow, pink, purple, red, burgundy) are shown at tips. The heat map shows the log of mean pigment mass fraction for the six anthocyanidins: pelargonidin (Pel), cyanidin (Cya), peonidin (Peo), delphinidin (Del), petunidin (Pet) and malvidin (Mal); and the three flavonols: kaempferol (Kae), quercetin (Que) and myricetin (Myr). ‘X’ indicates no detectable pigment. Totals are shown for both anthocyanins and flavonols; raw values are in electronic supplementary material, table S1. Pigment level distributions are in electronic supplementary material, figure S2. Representative flower images for each clade from top to bottom and left to right are as follows (with credits): *Fabiana punensis*, *Calibrachoa eglandulata*, *Petunia reitzii*, *Brunfelsia lactea*, *Nierembergia scoparia* (all by L. C. Wheeler), *Bouchetia erecta* (Edith Bergquist), *Hunzikeria texana* (Karla M. Benítez), *Plowmania nyctaginoides* (R. Deanna), *Nierembergia scoparia* (L. C. Wheeler), *Leptoglossis albiflora* (R. Deanna).

In order to explore the role of variation in gene expression in generating colour diversity, we focus on the Petunieae, a clade of roughly 180 species comprising the South American genus *Petunia* and eight allied genera. This group is widely known for its tremendous diversity in flower colours, including white, yellow, pink, purple and red (figure 1). Moreover, the cultivated petunia has long served as the premier system for studying the genetics and regulation of flower colour [26]. Importantly, studies in petunia as well as other taxa have demonstrated that many steps in the anthocyanin pathway are jointly regulated by a complex comprising R2R3 MYB, basic helix–loop–helix (bHLH) and WD40 transcription factors [27], allowing for coordinated expression of enzymes and the compounds they produce. In addition to anthocyanin pigments, *Petunia* flowers also produce UV-absorbing flavonols, which share biochemical precursors with anthocyanins but appear to be independently regulated by different R2R3 MYBs [28]. Changes in the expression of these transcription factors and in turn their downstream targets (pathway enzymes) underlie the loss of floral anthocyanins [29], the gain of floral UV patterning due to flavonols [28], and the shift to red anthocyanin pigmentation [30] in different *Petunia* species. We predict that this connection between pathway gene expression and pigment variation holds across the broader Petunieae clade and may explain its diversity of colours, including those beyond the range of variation observed in *Petunia* itself.

Although a number of comparative studies have related flavonoid profiles to macroevolutionary flower colour variation (e.g. [31–33]), our study encompasses the broadest quantitative analysis connecting such biochemical variation to patterns of gene expression across the flavonoid pathway. Using these transcriptomic data from 60 species, we first estimate patterns of co-expression between pathway enzymes and the previously characterized classes of transcriptional regulators in *Petunia*. Next, we apply morphospace approaches to characterize the pigmentation space of Petunieae and identify clusters within that space. Finally, we combine these datasets to determine how changes in gene expression associate with the major axes of variation in pigment production. Our results demonstrate that coordinated shifts in gene expression strongly predict repeated transitions from pale to intensely pigmented phenotypes and from the production of the common blue pigments to the less common red and purple pigments. These coordinated changes in gene expression also mediate sharp trade-offs between anthocyanins and flavonols, implicating an underappreciated role of these colourless compounds in shaping visible colour diversity. Overall, these findings show that the structure of the pathway plays a fundamental role determining the accessibility of pigment phenotypes and in turn shapes the evolutionary trajectories taken to reach distinct floral pigmentation phenotypes.

## 2. Methods

### (a) Transcriptome assembly

We generated RNA-seq data for corolla tissue from developing floral buds equivalent to *Petunia* bud stage 5 [34], with three replicates per species. The first replicate was the data used in [35], while the second and third replicates were generated using RNA extracted from the buds of additional individuals collected with the same voucher (time and location) as the first replicate. We generated RNA-seq libraries using the Illumina TruSeq kit with IDT-for-Illumina indexes and sequenced them on an Illumina NovaSeq 6000 instrument at the Weill Cornell Genomics Core Facility. For each species we combined the paired-end reads from all three replicates to increase depth of coverage. To assemble *de novo* transcriptomes for the 59 Petunieae species and the *Browallia americana* outgroup used in this study, we followed the pipeline from Wheeler *et al.* [35]. Briefly, the pipeline carries out the following steps: (i) trim the reads using IDT-for-Illumina adapter sequences, (ii) perform *de novo* transcriptome assembly using Trinity, (iii) detect and remove chimeric sequences using the run_chimera_detection.py script from Yang & Smith [36], (iv) run Corset to cluster and collapse transcripts and (v) predict CDS using TransDecoder.

### (b) Quantification of gene expression

We retrieved flavonoid pathway genes and their transcription factor regulators from transcriptomic CDS following the pipeline from Wheeler *et al.* [35]. Briefly, we used BLASTN to identify sequences matching queries (*e*-value cut-off = 1 × 10^−50^) for the structural genes: CHS-A, CHI-A, CHI-B, F3H, FLS, F3′H, F3′5′H, DFR, ANS, MF1, MF2 and MT; the transcription factors AN2, DPL, PHZ, AN11, AN1, JAF13, MYBFL, MYB27, AN4, ASR1, ASR2, ASR3; and the housekeeping genes actin, tubulin, Rps18, Gapdh, Hprt. We then filtered these hits by similarity to the query sequences (alignment score) using BioPython and removed all spurious sequences. For downstream analyses relating gene expression to pigment production, we included only the relevant pathway-related genes and transcription factors, excluding the housekeeping genes after examining them for quality control in preliminary analyses. By contrast to the approach taken previously, we did not reduce the BLAST hits to a single best match for each gene (see electronic supplementary material, text). Instead we combined paralogous transcripts (e.g. CHS-A, CHS-J) into a single collective fasta reference file. Because the subgroup 6 MYB activators (AN2, AN4, DPL, PHZ, ASR1, ASR2, ASR3) are functionally similar and individual gene presence in the transcriptomes varies considerably, we also combined this set of sequences into a single group SG6-Mybs (see electronic supplementary material, text). To confirm the accuracy of our gene extraction pipeline we performed a reverse BLASTN search of all the resulting sequences against the annotated CDS from the *Petunia inflata* genome v.1.0.1. To quantify gene expression we pseudo-mapped the reads from each individual replicate separately to the combined *de novo* transcriptome assembly of the corresponding species using Salmon v.1.5.2 [37]. To extract expression levels for the flavonoid pathway genes, we used the transcript IDs from the combined fasta reference files to parse the Salmon quant.sf files and then calculated a sum of expression levels for each gene by adding together the TPM values for all corresponding transcripts (e.g. CHI-A and CHI-B). We then normalized the resulting summed TPM values to TPM10K using the approach of Munro *et al.* [38], which accounts for the number of transcripts in each transcriptome. Scripts to conduct this analysis are available in electronic supplementary material, repository (https://osf.io/zg9cu/).

### (c) Quantification of anthocyanin and flavonol content

We used the same high-performance liquid chromatography (HPLC) approach to quantify the mass fraction of flavonoids as in our previous Petunieae work [35], following Berardi *et al.* [30]. With the exception of a few samples that were re-run for improved data quality, the anthocyanin mass fraction data are the same as that used to calculate average total pigment concentration for the species in [35]. However, we subsequently collected data for the flavonols (kaempferol, quercetin and myricetin) in corolla tissue of all replicate individuals using a similar approach. To ensure that anthocyanin and flavonol measurements were directly comparable, we conducted the flavonol measurements on the flavonol-containing layer remaining from the extraction procedure used to measure anthocyanin content. We sampled flowers from three individuals per species and used these to calculate the mean anthocyanin mass fraction (milligrams of compound per gram of tissue) over replicates, based on comparison with standard curves. For each individual, we collected fresh floral corolla tissue, dried the tissue with silica gel and stored the material in 2 ml tubes at −80°C. For extraction of total flavonoids, we soaked 0.002–0.75 g of dried tissue overnight in 1 ml 2 N HCl; more tissue was used for pale and fleshy species like *Brunfelsia* and less for thin and intensely coloured species like *Petunia*. We carried out acid hydrolysis of flavonoid glycosides and analysed the samples using HPLC as in [35]. Briefly, we heated samples 100–104°C for 1 h to convert the glycosylated flavonoids into their corresponding aglycones and then performed a series of liquid phase extractions in ethyl acetate and isoamyl alcohol, before evaporating away excess solvent using an N-EVAP apparatus and eluting in 50 µl of 1% HCl in MeOH. We injected 10 µl of sample on the Agilent HPLC and separated flavonols by gradient elution on a 100–4.6 mm Chromalith Performance column at 30°C using solvents A (HPLC-grade water, 0.1% trifluoroacetic acid) and C (methanol, 0.05% TFA). We analysed all results using Agilent Chemstation software and compared peaks to standards obtained from Extrasynthese (365 nm for flavonols and 520 nm for anthocyanidins) to calculate milligrams of pigment per extraction. We then normalized these milligrams of mass values by total dry mass of flowers (grams) to obtain the milligram per gram mass fraction for each pigment in each sample. Chemstation peak tables were individually cross-checked against chromatograms and manually corrected for slight peak shifts as needed.

### (d) Reconstruction of species phylogeny

We previously followed the approach of Walker *et al.* [39] to reconstruct the species tree for the Petunieae clade using 3672 orthologue clusters identified from the original *de novo* transcriptome assemblies as in [35]. However, for the current study, we added an additional species; *Fabiana australis* (four-letter code=PEPA), which has recently been renamed from *Petunia patagonica* [40]. To add *F. australis* into the analysis we started with the orthologue clusters from the previous publication (downloadable from https://osf.io/b7gcp/). We identified the best-matched sequence in the new *F. australis* transcriptome using BLASTN (*e*-value cut-off = 1 × 10^−50^), added these sequences into the clusters, re-ran the cluster alignments using MAFFT, and then re-ran the species-tree analysis in Astral 5.7.8 using the updated clusters. We followed the TreePL smoothing approach used in [35] to ultrametricize the tree, using a subset of 11 genes present in all 60 species.

### (e) Phylogenetic principal components analysis

**Figure 2.**
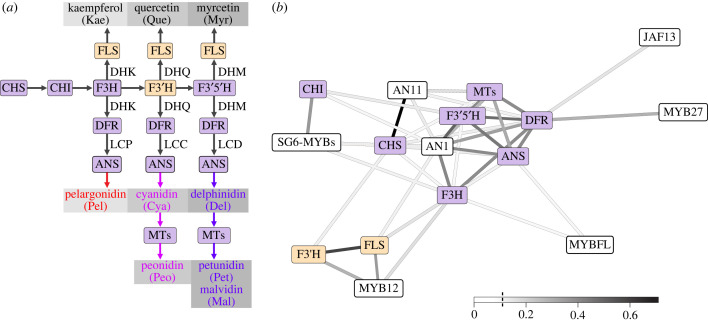
Two clusters of co-expressed pathway genes and transcription factors. (*a*) Simplified flavonoid pigment pathway, focusing on the major products found in Petunieae (the three flavonols and six anthocyanidins). Grey boxes around products indicate increasing levels of hydroxylation (left to right, mono-, di- and tri-hydroxylated). Key intermediates are abbreviated as follows: DHK, dihydrokaempferol; DHQ, dihydroquercetin; DHM, dihydromyricetin; LCP, leucopelargonidin; LCC, leucocyanidin; LCD, leucodelphinidin). Enzymes are shown in coloured boxes and coloured by their cluster in (*b*); see electronic supplementary material, table S2 for full gene names. Arrows show the small-molecule substrates/products passed from one enzyme to another. Three of the enzymes (FLS, DFR, ANS) are shown three times because they can potentially act on three different substrates (e.g. DHK, DHQ or DHM for DFR and FLS). (*b*) Correlation structure from the phylogenetic PCA of expression values for structural genes (coloured boxes) and transcription factors (white boxes). Positive values above the median (*R*^2^ > 0.124, indicated with a vertical line in the inset scale) were visualized with a force-directed spring layout representation. Edge weights (*R*^2^) are coloured by magnitude. See electronic supplementary material, figure S3 for full matrix of correlation coefficients. Distributions of gene expression levels are shown in electronic supplementary material, figure S4.

**Figure 3.**
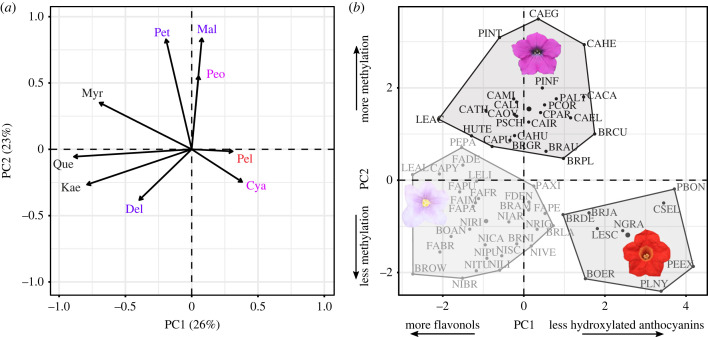
Clusters in pigment space defined by pathway branches. (*a*) Biplot from pPCA with flavonoids plotted by loading on the first two PC axes. Abbreviations follow figure 2. The three flavonols (quercetin, myricetin and kaempferol) plus the tri-hydroxylated delphinidin load negatively onto PC1 while the less hydroxylated pelargonidin and cyanidin load positively. The three methylated anthocyanidins (petunidin, malvidin and peonidin) load positively onto PC2. (*b*) Species of Petunieae plotted by values for PC1 and PC2. Taxon labels are coloured by k-means clustering. The flower of one species from each cluster is shown; taxon abbreviations follow figure 1. The convex hull of the points within each cluster is drawn with solid lines.

To more closely approximate normally distributed data, we transformed the pigment mass fraction (mg g^−1^) values by applying a ln(mg/g × 100 + 1) transformation and the gene expression (TPM10K) values by applying a ln(TPM10K + 1) transformation. We used the *phyl*.*pca* function from *phytools* [41] and the *prcomp* function from *stats* [42] in R v. 3.6.3 to perform a phylogenetic principal components analysis (pPCA) while scaling and centring the transformed data. To obtain the underlying correlation matrix between transformed TPM10K gene expression levels for all genes incorporated in the analysis, we extracted the covariance matrix from the PCA output (the *V* attribute) and used the *cov*2*cor*function to convert it to a matrix of correlation coefficients. To convert this matrix into the network shown in figure 2 we selected all positive correlation coefficients larger than the median value (0.124) and used *networkx* [43] in Python v.3.8.5 to convert the matrix to a graph edge list. We generated the network figure, with edges coloured according to weights (correlation coefficients) using Cytoscape v.3.9.1 [44]. To generate the pigment level clusters shown in figure 3, we performed k-means clustering on the first three principal components from the pigment pPCA using the *kmeans* function in R with three clusters, based on the ‘elbow method’ after plotting within-cluster mean-squared error as a function of the number of clusters.

### (f) Phylogenetic canonical correlation analysis

To assess the relationships between expression of flavonoid pathway-related genes and flavonoid pigment levels, we performed phylogenetic canonical correlation analysis (pCCA) on the transformed data using the $\ln([\mathrm{mg}\;g^{-1}]\times 100)+1$ function in the R *phytools* package. We treated the gene expression levels as the ‘*x*’ variable and pigment mass fraction as ‘*y*’. We used the *p*-values calculated by ln(TPM10K + 1) to determine the statistical significance of the canonical variates (CVs). We extracted the canonical coefficients from the significant CVs, which quantify the coupled associations of the original pigment mass fraction and gene expression variables with the corresponding multivariate CVs, and standardized them. We re-calculated the significant CVs, arrayed by species ID, as the linear combination of the original variables scaled by un-standardized coefficients. We then used the *phyl*.*pca* function in R to calculate each canonical loading (correlation coefficients of original variables with their corresponding CV) and cross-loading (correlation coefficients of original variables with the CV for the other data block; e.g. pigment levels with gene expression CV1) with corresponding *p*-values.

### (g) Stochastic mapping and ancestral state estimation

We used the stochastic mapping tools in phytools to estimate the number of transitions between each pigment phenotype from the k-means clustering of pPC scores. We carried out 200 realizations using the make.simmap in *phytools*. We used an equal rates model, as the all-rates-different model did not provide a significantly better fit to the data according to a likelihood ratio test. We summarized the 200 realizations to obtain estimated ancestral states at each node.

### (h) Molecular evolution

We selected a set of structural genes and transcription factors that were present in the majority of taxa: AN1, AN11, ANS, CHI-A, CHS-A, DFR, F3H, F3′H, F3′5′H, FLS, JAF13, MT and MYB27. We extracted a single sequence, best-matched to the query sequence for each gene, from each species using the approach of Wheeler *et al.* [35]. In this analysis, we excluded the subgroup 6 MYB transcription factors due to their absence in the *de novo* transcriptomes of many species in the dataset. A previous analysis showed that these MYBs did not present patterns consistent with adaptive substitutions related to flower colour transitions [35]. We used HyPhy to fit a free-rates d*N*/d*S* model that allows a separate d*N*/d*S* ratio for each tip. We then extracted d*N*/d*S* trees from the HyPhy output and calculated a root-to-tip d*N*/d*S* ratio for each tip. We assessed the relationships between these values and the principal axes of flavonoid variation using linear regression (for details see electronic supplementary material, text).

## 3. Results

### (a) Flower colour diversity is matched by diversity of pigment profiles

Species of Petunieae produced all six types of anthocyanidins, the base molecules that are modified to form glycosylated anthocyanins, and all three classes of the flavonol co-pigments. Delphinidin and its two methylated forms (petunidin and malvidin), commonly associated with blue and purple flowers [23], are the most commonly produced pigments while the other three classes of pigments are only found in a few species (figure 1, [45]). The total quantity of anthocyanin pigments varies widely across species, with the many white-flowered species, like *Nierembergia rigida*, producing little to no anthocyanins and the deep purple and pink-flowered species, like *Calibrachoa caesia*, producing over 3 mg g^−1^ petal tissue (figure 1; see also [35]). Some predominantly white-flowered species, such as *Calibrachoa ovalifolia* and *C. pygmaea*, also produce relatively high amounts of anthocyanins, due to pigmentation of the floral veins (figure 1, electronic supplementary material, table S1). Petunieae flowers of all colours produce abundant flavonols, often at levels that are orders of magnitude higher than the anthocyanins (figure 1, electronic supplementary material, table S1). These compounds may act as co-pigments, altering hue or intensifying the colour [46] and/or contributing to UV patterning involved in pollinator attraction [28].

### (b) Phylogenetic correlation structure reveals co-expression relationships across the flavonoid pathway

We used petal transcriptomic data for 59 Petunieae species to examine clade-wide patterns of co-expression among nine enzymes and seven transcription factors of the flavonoid pathway. For this and subsequent analyses, we grouped two sets of genes, the methyl-transferases (MTs) and R2R3 MYB subgroup 6 activators, which vary in copy number across taxa but carry out similar functions (see electronic supplementary material, text). We computed correlation coefficients, accounting for phylogenetic structure, and found two clusters of correlated structural genes, a flavonol module (F3′H and FLS) and an anthocyanin module, comprising the remaining steps of the pathway (figure 2). The ‘late’ anthocyanin biosynthesis (F3′5′H, DFR, ANS and the MTs) form a tight cluster while the other core pathway genes (CHS and CHI) are more loosely connected. As expected, the components of the MBW complex (the SG6 MYBs, the bHLH AN1 and the WD40 AN11) are mostly strongly associated with the anthocyanin module, while the flavonol regulator MYB12 [47] is co-expressed with the flavonol module. Another flavonol regulator, MYB-FL, was not co-expressed with the flavonol module, suggesting its role may be specific to the clade of *Petunia* in which it was studied [28]. We also found the repressor MYB27 is most associated with DFR expression, consistent with the notion that it is upregulated after the late steps in the pathway to provide feedback inhibition [14]. The tighter connection of AN1 to anthocyanin biosynthesis compared to the other bHLH transcription factor (JAF13) may relate to the relatively late bud stage sampled; the two bHLH genes are functionally similar but AN1 acts later in floral development [14,48].

### (c) Pigment phenotypes are divided by hydroxylation, methylation and flavonoid content

A pPCA of pigment production (figure 1) revealed sharp trade-offs among pathway branches, as manifested in the pigment profiles across species. The first PC axis, which accounts for 26% of the variation, is driven by the level of hydroxylation and the amount of flavonol production (figure 3, electronic supplementary material, table S3). It separates pale-flowered species, which produce the tri-hydroxylated delphinidin and high amounts of flavonols, from those which produce the less hydroxylated cyanidin and pelargondin and lower amounts of flavonols, including the bright red-flowered *Plowmania nyctaginoides* and *Petunia exserta* (PLNY, PEEX). The intensely coloured purple and pink-flowered species characteristic of *Petunia* and *Calibrachoa* are intermediate along this axis, with mostly tri-hydroxylated anthocyanins and a range of flavonol concentrations. The second PC axis reflects the level of methylation and divides the taxa that produce the unmethylated anthocyanidins (delphinidin, cyanidin, pelargonidin) from those that produce mostly or entirely methylated compounds (peonidin, petunidin, malvidin). We used k-means clustering to group to the taxa in this pigment profile space and recovered three clusters, the pale-flowered taxa making large amounts of flavonols, the deeply pigmented taxa making methylated anthocyanidins, and the taxa making less hydroxylated anthocyanidins and lower flavonols. While the first two clusters are fairly uniform in colour (white to light purple and deep pink to deep purple, respectively), the cluster containing the diverse less hydroxylated anthocyanins and low flavonols range in colour from yellow (BRDE, LESC) to pink (PBON, CSEL) to red (PLNY, PEEX). In the absence of the yellowish flavonols, the yellow hues in these taxa are likely derived from floral carotenoids (visible under light microscopy, SD Smith 2020, unpublished data).

### (d) Pathway gene expression predicts major pigment phenotypes

**Figure 4.**
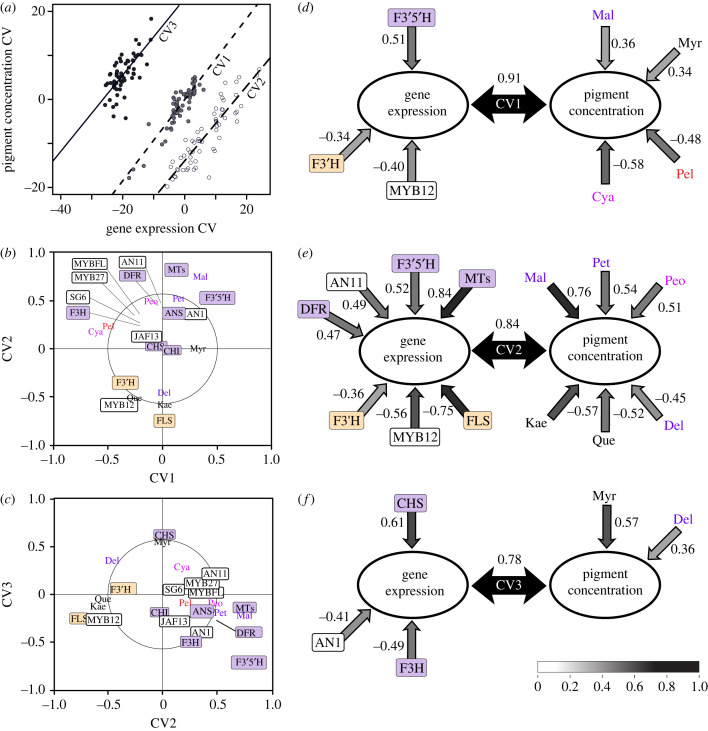
Pathway gene expression correlates tightly with pigment production. (*a*) Scatterplot of the significant CVs for pigment concentration and gene expression from phylogenetic canonical correlation analysis (pCCA). The correlation coefficients for each gene expression CV and pigmentation CV are shown in (*d*–*f*), inset in the black arrows. (*b*,*c*) Biplots of loadings of original expression and pigment variables onto CVs. For some tightly clustered variables, the location of their point is indicated with a line. (*d–f*) Variables with significant loadings onto each CV. Pearson correlation coefficients are shown for each significant variable (expression level or pigment amount) with one-way arrows. The bidirectional black arrows show the strength of the correlation between the given expression and pigment CVs.

pCCA revealed a tight relationship between the expression of flavonoid pathway structural genes and regulators, and the production of flavonoid compounds. The first three CVs are statistically significant and have strong correlations between gene expression and pigment concentration variables (figure 4). Biplots of loadings for each gene and pigment on each CV (electronic supplementary material, tables S4 and S5) show similar clustering patterns as recovered in the individual analyses. For example, the flavonol module corresponding to F3′H, FLS and MYB12 (figure 2) emerges from the pCCA (figure 4*b*,*c*) and is associated with the two flavonols showing correlated production, quercetin and kaempferol (figure 3). Similarly, the three methylated anthocyanidins (peonidin, petunidin and malvidin) are associated with several of the late pathway genes (F3′5′H, ANS, MT) that control their production (figure 4*b*). Moreover, the CVs explain the expression variation underlying the major axes of pigment variation identified in the pPCA (figure 3). The first CV identifies genes whose expression contributes to hydroxylation level, which distinguishes the red-flowered species from the rest. Specifically, production of the less hydroxylated pelargonidin and cyanidin is correlated with high expression of F3′H and its regulator MYB12 and low expression of F3′5′H (figure 4*d*), which diverts production towards the tri-hydroxylated compounds (figure 2*a*). The second CV explains the production of flavonols and methylated anthocyanins (figure 4*e*). Here, high expression of the MTs and other late pathway genes leads to high levels of the methylated anthocyanins responsible for the intense purples and pinks as in most *Petunia* and *Calibrachoa*. Conversely, high expression of the flavonol module shifts production away from anthocyanins and toward the flavonols quercetin and kaempferol, as observed in the pale and white-flowered species. Finally, the third CV addresses production of the most common anthocyanidin across the species, delphinidin, and its flavonol counterpart, the tri-hydroxylated myricetin. Their production appears to be shaped by expression of early genes in the pathway, which control overall flux [49].

### (e) Relationship between pigment types and genes not broadly driven by functional evolution

Changes in coding sequences may also contribute to the relationship between particular enzymes and pathway outputs (e.g. [22]). For example, we might expect relaxed selection on F3′5′H in lineages that have moved away from the production of tri-hydroxylated anthocyanins [50]. Similarly, the MTs would be predicted to experience strong purifying selection in the clades with high production of methylated anthocyanins. We tested for relationships between the rates of non-synonymous to synonymous substitutions (d*N*/d*S*) across the pathway genes and major axes of pigment variation (total anthocyanins, total flavonols, fraction methylated anthocyanins, fraction tri-hydroxylated delphinidin derivatives). Despite wide variation in d*N*/d*S* across genes (see [35] for an in-depth discussion), we recovered no significant correlations between root-to-tip rates and pigment phenotypes (electronic supplementary material, table S6 and figures S8–S11). These results suggest that changes at the coding level are not the primary drivers of pigment variation across the species.

Nevertheless, we expect that high levels of red pelargonidin pigments should be limited by the inability of *Petunia* DFR to reduce the precursor dihydrokaempferol [51]. Therefore, we examined the DFR sequence in *Pl. nyctaginoides*, the only species found to produce primarily pelargonidin (figure 1). Compared with other sequenced Petunieae species, this species has a unique Q226K substitution (relative to *Vitis vinifera* sequence positions in crystal structure 2c29) in the active site, which would be in close contact with the substrate (electronic supplementary material, figure S4). This precise substitution has also been documented in a distantly related red-flowered pelargonidin-producing Solanaceae species and it has been shown to increase DFR activity on DHK [22]. Interestingly, all three sequenced *Pl. nyctaginoides* individuals carry both the Q (CAA) and K (AAA) codons at this position, suggesting that either all are heterozygous, or that there are two nearly indistinguishable DFR copies in this species (electronic supplementary material, figure S5 and text). All individuals are fixed for a substitution Y227F, which is shared by close relatives *Bouchetia* and *Hunzikeria* (as well as *V. vinifera*) but absent in other Petunieae species. Given its close proximity to the Q226K substitution and its presence in the active site, it is possible that Y227F interacts with Q226K to change the active site environment and may have played a role in a shift in DFR function in *Pl. nyctaginoides*.

### (f) The deeply pigmented phenotypes are likely derived from the pale colours

**Figure 5.**
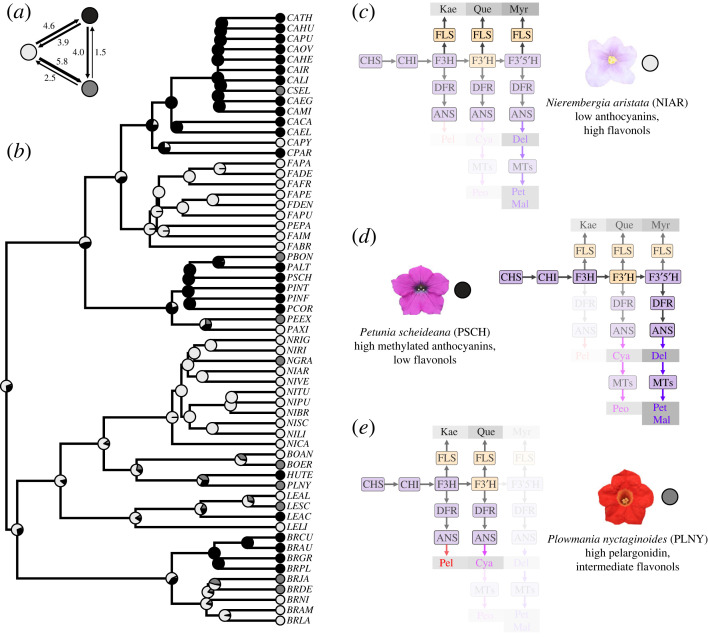
Deep purple and red colours may have evolved from pale ancestors. (*a*) Estimated numbers of transitions between each pigment phenotype from stochastic mapping. (*b*) Ancestral state estimation of the three pigmentation clusters (shown in figure 3*b*) from stochastic mapping. The outgroup (*Browallia americana*) was pruned from the tree to better visualize nodes inside Petunieae. (*c–e*) Exemplar species from each cluster. Steps of the flavonoid pathway and pathway products (figure 2*a*) are shaded by their expression in each, with the lower expressed branches being least visible.

We used the phylogeny to estimate the evolutionary history of the major pigment phenotypes in Petunieae. Using the best-fitting equal rates model and the pigment states from the pPCA (figure 3), we infer that the ancestor of Petunieae most likely belonged to the pale-flowered, delphinidin-producing, high flavonol phenotype (*p* = 0.7) with multiple transitions to the other phenotypes (figure 5*a*,*b*). This pale-flowered state has been retained in *Fabiana* and *Nierembergia*, as well as some *Brunfelsia* and is characterized by relatively low overall pathway expression, but high FLS expression, leading to high flavonol accumulation (figure 5*c*). The intensely coloured and highly methylated (electronic supplementary material, figure S6) pink-purple phenotype is characteristic of *Petunia* and *Calibrachoa*, while the lineages that have diverged to produce less hydroxylated anthocyanins and/or lower amounts of flavonols are scattered throughout the tree, arising from ancestors of both of the other states (figure 5*a*,*b*). The transition to producing high amounts of the tri-hydroxylated and methylated anthocyanins requires a shift to high expression of all pathway steps and typically comes at the expense of flavonol production (figures 4*e* and 5*d*). The red-flowered species producing less hydroxylated anthocyanins also tend to produce lower amounts of flavonols (figure 5*e*), a pattern observed in other Solanaceae [52], but which has not been broadly examined in other families.

## 4. Discussion

Our study revealed that Petunieae produce all of the six classes of anthocyanidins, including three main branches (the red pelargonidin, purple cyanidin and blue delphinidin pigments) and all three methylated derivatives (figure 1). Although most species present only delphinidin and its derivatives petunidin and malvidin, a few species are able to produce pigments down two or even three branches. The UV-absorbing flavonols are present in all species, but with concentrations varying over 1000-fold (electronic supplementary material, table S1 and figure S2). Through multivariate analyses of these biochemical profiles, we found that species are clustered in pigment space by the degree of hydroxylation and methylation of the anthocyanins and the extent of flavonol production. These axes of variation in pigment production are tightly correlated with variation in gene expression of the corresponding branches of the pathway, supporting the notion that regulatory changes are the principal drivers of flower colour evolution. Nevertheless, the relative rarity of species that have deviated from the ancestral state of making delphinidin and delphinidin-derived anthocyanins points to constraints in moving along the hue axis.

### (a) Evolutionary increases in pigment intensity coupled with higher methylation

Changes in the amount of anthocyanin production, whether associated with continuous variation in the intensity of coloration or discrete gains and losses of flower colour, are common throughout angiosperms [53]. Our phylogenetic analysis estimates four to five transitions to the intensely pigmented purple phenotype, in the large genera *Petunia*, *Calibrachoa* and *Brunfelsia* as well as in *Leptoglossis* and *Hunzikeria* (figure 5). These flowers range from hot pink, to magenta to purple, and at least for *Petunia* and *Calibrachoa* are bee-pollinated [54,55]. This increase in anthocyanins often comes at the cost of flavonols (figures 2*b* and 4), which could influence floral UV absorbance and in turn, pollinator preference (e.g. [56]). Nevertheless, given the abundance of bee pollination in *Petunia* and *Calibrachoa*, we expect that any flavonol production is concentrated in the centre to serve as bulls-eyes to enhance floral attraction [57]. We also found that the shift to producing high amounts of delphinidin-derived anthocyanins is reversible in Petunieae, and several of these lineages have subsequently transitioned to the two other pigment composition types (figure 5).

One unexpected finding of this study was that these convergent transitions to intense pigmentation involve not only increasing flux down the delphinidin branch, but increasing methylation as well (figure 3, electronic supplementary material, figure S6). This pattern may relate to the co-regulation of MTs with other late pathway genes (figure 2, [58]). If increases in floral pigmentation often occur via trans-regulatory mutations [24], the expression of MTs may be elevated together with F3′5′H, DFR and ANS, pulling flux toward petunidin and malvidin production. The predominance of methylated anthocyanins in highly pigmented flowers may also have effects on the colour phenotype and its stability. Methylation has a reddening effect on the bluish delphinidin pigments [59], which could contribute to the hot pink hues of many of these species. Moreover, methylation has important biochemical properties, increasing stability and water solubility [60,61]. These factors may be particularly important as the high levels of production of anthocyanins comes at the expense of flavonols (esp. quercetin and kaempferol, figure 4*e*), which can also stabilize anthocyanins through intermolecular stacking [62].

### (b) Limited evolutionary transitions in anthocyanin composition likely due to ancestral preference

Shifts in floral hue (e.g. from blue to pink) are often associated with changes in the type of anthocyanin produced. Specifically, transitions from blue or purple to red commonly involve shifting from more to less hydroxylated anthocyanins (reviewed in [23]). Despite the range of colours present in Petunieae (figure 1), we found that such changes in the level of hydroxylation are uncommon (see also [31,45]). Although 10 species make detectable amounts of pelargonidin and cyanidin (electronic supplementary material, table S1), these are generally present in trace amounts. The exceptions are *Pe. exserta*, which produces roughly half cyanidin and half delphinidin and methylated derivatives [30], and *Pl. nyctaginoides*, which makes 96% pelargonidin. The addition of carotenoids may further contribute to the intensity of the red coloration in *Pl. nyctaginoides*, but anthocyanins alone underlie the colour change in *Pe. exserta* (electronic supplementary material, figure S7, [30]). Our phylogenetic CCA suggests that the downregulation of F3′5′H is the most highly correlated expression difference associated with shifts away from the production of delphinidin-derived anthocyanins (figure 4*d*), a pattern observed in other clades where red flowers have evolved (e.g. [20,63,64]).

The fact that Petunieae present a range of pink, fuschia and purple hues despite largely producing only delphinidin-derived pigments (figure 1) implicates other mechanisms for diversifying colour. Combining anthocyanins with carotenoid pigments to produce redder hues is a common strategy in flowering plants (e.g. [65,66]), and several of the hot pink *Petunia* and *Calibrachoa* species express floral carotenoids (e.g. *Pe. correntina*, *C. caesia*, LC Wheeler & SD Smith 2022, unpublished data)). Acidification of the vacuole, where anthocyanins are stored, can also shift the colour to appear more red [59]. This phenomenon is known in cultivars of *Petunia* and *Calibrachoa* [67], but not yet documented as part of an evolutionary colour transition. Finally, in addition to the reddening effect of methylation mentioned above, acylation of anthocyanins has a blueing effect, so reduction in acylation can also contribute to redder colours [30,45]. The most deeply red *Calibrachoa*, *C. sendtneriana*, is extremely rare [68], and although we were not able to obtain replicates to include in the present study, previous work demonstrates that it only produces delphinidin derivatives [25], making it another Petunieae species to produce red flowers with blue pigments. Other Petunieae with unique shades, such as the bright salmon-coloured *Petunia reitzii* and the burgundy *Leptoglossis acutiloba* also comprise candidates for using a combination of biochemical mechanisms to produce diverse colours.

The rarity of shifts from producing delphinidin-derived anthocyanins to those derived from pelargonidin also points to strong underlying constraints in moving along the hydroxylation axis. The most likely source of such constraints is substrate specificity of multi-functional pathway enzymes (e.g. DFR, ANS, figure 2*a*). The inefficiency of *Petunia hybrida* DFR in acting on pelargonidin precursors has been well documented as part of efforts to breed red horticultural varieties (e.g. [69–71]). The prevalence of delphinidin-derived anthocyanins across the Petunieae suggests that the preference for the precursors of delphinidin is not particular to *Pe. hybrida*, but likely represents the ancestral state for the clade, and perhaps for the entire Solanaceae [22]. In this context, it is notable that the only species of Petunieae to make predominantly pelargonidin, *Pl. nyctaginoides*, carries the precise single amino acid mutation found in another red-flowered lineage of Solanaceae which is known to more than double activity on the pelargonidin precursor, dihydrokaempferol (electronic supplementary material, figure S5, [22]). These patterns suggest that transitioning to pelargonidin production is accessible only through changes in the ancestral enzyme function.

## 5. Conclusion

Comparative evo-devo studies have the potential to reveal commonly traversed evolutionary paths and the mechanisms underlying those phenotypic shifts. Floral pigmentation has long been the subject of comparative analysis in plants (e.g. [72–74]), allowing us to identify those frequently travelled evolutionary paths (e.g. from blue to white coloration) and laying the groundwork for connecting these transitions to changes in the expression and function of the biochemical pathways. Our study demonstrates that *Petunia* and its close relatives have diversified in pigmentation by repeatedly calibrating the production of blue delphinidin-derived pigments and UV-absorbing flavonols through changes in gene expression in the anthocyanin pathway. We posit that these axes comprise evolutionary paths of least resistance, whereby adjusting gene expression allows for a wide range of visible and UV–visible pigmentation levels. However, expression changes are probably insufficient to overcome ancestral patterns of substrate specificity in multi-functional enzymes to allow transitions along the hydroxylation axis. Thus, moving beyond the range of colours accessible by changing anthocyanin and flavonol levels alone likely requires novel mutations to enzyme activity and/or the recruitment of additional biochemical tricks, such as vacuolar acidification, decoration of anthocyanins with acyl groups, or co-expression with carotenoids, to reach new colour phenotypes.

## Data Availability

The transcriptome assemblies, scripts and processed data files used to conduct the analyses are available in the supplementary OSF repo (https://osf.io/zg9cu/). The raw RNA-seq data files have been added to SRA BioProject PRJNA746328. Additional information is provided in the electronic supplementary material [75].
